# Validation of the SCID-hu Thy/Liv Mouse Model with Four Classes of Licensed Antiretrovirals

**DOI:** 10.1371/journal.pone.0000655

**Published:** 2007-08-01

**Authors:** Cheryl A. Stoddart, Cheryl A. Bales, Jennifer C. Bare, George Chkhenkeli, Sofiya A. Galkina, April N. Kinkade, Mary E. Moreno, José M. Rivera, Rollie E. Ronquillo, Barbara Sloan, Paul L. Black

**Affiliations:** 1 Gladstone Institute of Virology and Immunology, University of California at San Francisco, San Francisco, California, United States of America; 2 Targeted Interventions Branch, Division of AIDS, National Institute of Allergy and Infectious Diseases, National Institutes of Health, Bethesda, Maryland, United States of America; AIDS Research Center, Chinese Academy of Medical Sciences and Peking Union Medical College, China

## Abstract

**Background:**

The SCID-hu Thy/Liv mouse model of HIV-1 infection is a useful platform for the preclinical evaluation of antiviral efficacy *in vivo*. We performed this study to validate the model with representatives of all four classes of licensed antiretrovirals.

**Methodology/Principal Findings:**

Endpoint analyses for quantification of Thy/Liv implant viral load included ELISA for cell-associated p24, branched DNA assay for HIV-1 RNA, and detection of infected thymocytes by intracellular staining for Gag-p24. Antiviral protection from HIV-1-mediated thymocyte depletion was assessed by multicolor flow cytometric analysis of thymocyte subpopulations based on surface expression of CD3, CD4, and CD8. These mice can be productively infected with molecular clones of HIV-1 (e.g., the X4 clone NL4-3) as well as with primary R5 and R5X4 isolates. To determine whether results in this model are concordant with those found in humans, we performed direct comparisons of two drugs in the same class, each of which has known potency and dosing levels in humans. Here we show that second-generation antiretrovirals were, as expected, more potent than their first-generation predecessors: emtricitabine was more potent than lamivudine, efavirenz was more potent than nevirapine, and atazanavir was more potent than indinavir. After interspecies pharmacodynamic scaling, the dose ranges found to inhibit viral replication in the SCID-hu Thy/Liv mouse were similar to those used in humans. Moreover, HIV-1 replication in these mice was genetically stable; treatment of the mice with lamivudine did not result in the M184V substitution in reverse transcriptase, and the multidrug-resistant NY index case HIV-1 retained its drug-resistance substitutions.

**Conclusion:**

Given the fidelity of such comparisons, we conclude that this highly reproducible mouse model is likely to predict clinical antiviral efficacy in humans.

## Introduction

The SCID-hu mouse model, in which human lymphoid organs are implanted into severe-combined immunodeficient (SCID) mice, was designed by McCune et al. [1] to provide a small animal model for the study of human hematopoiesis. It has also facilitated study of the pathogenesis of HIV-1 in human hematolymphoid organs [2]–[7] and evaluation of anti-HIV-1 compounds *in vivo* [2], [8], [9]. In this model, SCID mice are implanted with a variety of human fetal organs, including bone, liver, thymus, lymph node, and spleen. The fetal implants become tolerant of the mouse environment, and reciprocally, growth of the human tissue is permitted by the immunocompromised status of the recipient SCID mouse [6].

The SCID-hu Thy/Liv mouse, first reported by Namikawa et al. in 1990 [10], is generated by coimplanting human fetal thymus and liver beneath the mouse kidney capsule. In a highly reproducible manner, these organs fuse, become vascularized, and grow into a stable organ termed “Thy/Liv,” reaching a total mass of 100–300×10^6^ human cells in 18 weeks. The Thy/Liv implant reproduces the differentiation, proliferation, and function of human hematopoietic progenitor cells derived from the fetal liver within the human thymus. The implants possess histologically normal cortical and medullary compartments that sustain multilineage human hematopoiesis for 6–12 months [10], [11], generating a continuous source of CD4-expressing thymocytes that can serve as target cells for HIV-1 infection and replication. Importantly for a model of antiviral chemotherapy, 50–60 SCID-hu Thy/Liv mice can be made with tissues from a single fetal donor, and the Thy/Liv implant is amenable to experimental manipulation and infection with HIV-1.

The Thy/Liv implants support viral replication after inoculation of HIV-1 by direct injection [3], and thymocyte depletion occurs with both molecular clones and clinical isolates of HIV-1 in 3–5 weeks [12]–[16]. This depletion includes loss of CD4^+^CD8^+^ (double-positive, DP) immature cortical thymocytes and a decrease in the CD4/CD8 ratio in the thymic medulla. There is evidence for both indirect, apoptotic destruction of uninfected thymocytes and direct infection and destruction of CD3^−^CD4^+^CD8^−^ intrathymic T-progenitor cells, which severely disrupts thymocyte maturation [17], [18]. Infection of human thymus with HIV-1 induces IFN-α secretion from plasmacytoid dendritic cells, which upregulates MHC class I (MHC-I) expression on DP thymocytes, where expression is normally low [19].

The administration of nucleoside and nonnucleoside reverse transcriptase (RT) inhibitors to these mice results in dose-dependent inhibition of HIV-1 replication, prevention of IFN-α-mediated MHC-I upregulation, and protection of CD4^+^ cells within the implanted human tissue [8], [9], [20]. The model has been also used to evaluate new classes of HIV-1 inhibitors, such as bicyclam [21] and oligonucleotide [22] inhibitors of HIV-1 entry, the nucleoside analog 2′-deoxy-3′-oxa-4′-thiocytidine (dOTC) [20], and an oxime-piperidine CCR5 antagonist [23]. This is the first animal model in which the action of candidate anti-HIV-1 compounds can be tested within the setting of an intact HIV-1-infected human target organ.

We use a standardized protocol described by Rabin et al. [8] for evaluation of antiviral agents against HIV-1 in the SCID-hu Thy/Liv mouse model. Drugs can be administered by various routes (oral, subcutaneous, intraperitoneal, or by mini-osmotic pump) before or after virus inoculation and continued for 2–6 weeks. Mice are challenged with titrated inocula of various HIV-1 isolates, and endpoint analyses include quantification of viral protein by p24 ELISA and viral RNA by branched DNA assay, quantification and isolation of replication-competent virus by cocultivation after limiting dilution, and assessment of the effects on human thymocyte subpopulations by flow cytometry [8].

The SCID-hu Thy/Liv model has also been employed by us and by others for *in vivo* studies of HIV-1 pathogenesis. The roles of HIV-1 *nef* [24]–[26], other accessory genes [24], and the Rev–Rev responsive element [27] have been explored as well as the expression of HIV-1 coreceptors (CXCR4 and CCR5) on thymocytes [28], [29] and the effects of coreceptor usage on viral tropism and pathogenesis [16], [28], [30]–[32]. The CXCR4-utilizing (X4) strain NL4-3 replicates quickly and extensively in thymocytes in the cortex and medulla, causing significant thymocyte depletion [16]. In contrast, the CCR5-utilizing (R5) strain Ba-L initially infects stromal cells, including macrophages, in the thymic medulla without any obvious pathologic consequences for 3–4 weeks, after which the infection slowly spreads through the thymocyte populations and results in slowly progressing thymocyte depletion after 6 weeks. In addition, three R5 viruses isolated from rapid AIDS progressors lacking X4 strains were much less pathogenic than NL4-3 in SCID-hu Thy/Liv mice, suggesting that R5 virus-mediated rapid disease progression is associated with host, not viral, factors [30]. The lab-adapted LAI/IIIB isolate and its associated infectious molecular clones (e.g., HXB2) fail to replicate in Thy/Liv implants, and this impairment has been mapped to a specific amino acid change in the crown region of V3 in Env [33], [34]. The model has also been used to study immune reconstitution and recovery of hematopoietic colony-forming activity in HIV-1-infected implants after treatment of mice with potent combination antiretroviral therapy [35]–[37].

Given the potential correlations between virus-host interactions in the Thy/Liv implant and in humans, we wanted to determine whether the SCID-hu Thy/Liv mouse model predicts antiretroviral efficacy in humans for representatives of all four classes of currently licensed drugs. Does it, for instance, yield data that reflect the greater potency of more recently developed drugs than their first-generation predecessors in each class? *In vivo* models are far more stringent than cell culture-based assays for demonstrating drug activity, particularly in the case of orally delivered drugs as they must be absorbed by the gastrointestinal tract without degradation, enter the blood and circulate with sufficient half-lives, and penetrate solid target organs at concentrations high enough to be potently efficacious. The greater potency of the second-generation drugs is likely the result of a combination of greater *in vitro* potency and superior pharmacokinetics. *In vitro* virus-cell culture systems would likely predict greater potency, but only *in vivo* studies can demonstrate sustained plasma half-life or superior oral bioavailability. To determine such relative potency *in vivo*, head-to-head comparisons of two drugs in the same class were performed in SCID-hu Thy/Liv mouse cohorts each made with tissues from a single donor. We observed that the more recent drugs in each class were significantly more potent than their predecessors. We conclude from such comparisons with licensed antiretrovirals that this well-established model is likely to predict clinical antiviral efficacy in humans for other members of these classes and possibly other drug classes as well.

## Materials and Methods

### Viruses

The following reagents were obtained through the AIDS Research and Reference Reagent Program, Division of AIDS, NIAID, NIH: pNL4-3 [38] from Malcolm Martin and HIV-1 Ba-L [39] from Suzanne Gartner, Mikulas Popovic, and Robert Gallo. A working stock of NL4-3 was prepared in 293T cells by calcium phosphate transfection. A T-20-sensitive NL4-3 (NL4-3 D36G) was altered by site-directed mutagenesis to match the consensus sequence at amino acid position 36 (aspartic acid replaced by glycine) [40], [41]. The R5X4 AZT-resistant clinical HIV-1 isolate JD [42] was kindly provided by Mike McCune, and the multidrug-resistant (MDR) R5X4 NY index case isolate [43] by Hiroshi Morhi and Martin Markowitz. The clinical isolates were prepared and titrated by limiting dilution in phytohemagglutin-activated peripheral blood mononuclear cells.

### Antiretroviral drugs and mouse dosing

The antiretroviral drugs lamivudine (3TC), emtricitabine [(–)-FTC], nevirapine, efavirenz, indinavir, and atazanavir were kindly supplied by Opendra Sharma and the AIDS Reagent Program. Dosing solutions were prepared in sterile water for injection or Dulbecco's phosphate-buffered saline, and groups of 5–8 mice were treated by oral gavage (200 µl per dose) with an 18-gauge×2-inch feeding needle twice daily at 7:00 AM and 6:00 PM for the duration of the infection period (2–6 weeks depending on the HIV-1 isolate). Enfuvirtide (T-20) was purchased from a pharmacy, prepared in sterile water, and administered by twice-daily subcutaneous injection (200 µl per dose).

### Implantation and inoculation of SCID-hu Thy/Liv mice

Human fetal thymus and liver were obtained through services provided by a nonprofit organization (Advanced Bioscience Resources, Alameda, CA) in accordance with federal, state, and local regulations. Coimplantation of thymus and liver fragments under the kidney capsule to create SCID-hu Thy/Liv mice and inoculation of the Thy/Liv implants with HIV-1 was carried out as described [8], [10]. Male C.B-17 SCID (model #CB17SC-M, homozygous, C.B-Igh-1^b^/IcrTac-Prkdc^scid^) mice were obtained at 6–8 weeks of age from Taconic (Germantown, NY), and cohorts of 50–60 SCID-hu Thy/Liv mice were implanted with tissues from a single donor. Implants were inoculated 18 weeks after implantation with 50 µl of stock virus (500–2,000 TCID_50_) or RPMI 1640 medium (mock infection) by direct injection after surgical exposure of the implanted kidney of anesthetized mice. Animal protocols were approved by the UCSF Institutional Animal Care and Use Committee.

### Implant collection and viral load quantification

The Thy/Liv implants were collected from euthanized mice, and single-cell suspensions were prepared by dispersing the implant through nylon mesh and processed for p24 ELISA, bDNA assay, and FACS analysis as described [8], [20]. Unless specified otherwise, implants were collected 21 days after inoculation with NL4-3, 14 days after JD inoculation, and 42 days after Ba-L inoculation.

### Flow cytometry

Implant cells were stained with phycoerythrin cyanine dye CY7-conjugated anti-CD4 (BD Biosciences, San Jose, CA), phycoerythrin cyanine CY5.5-conjugated anti-CD8 (Caltag Laboratories, Burlingame, CA), allophycocyanin cyanine CY7-conjugated anti-CD3 (eBiosciences, San Diego, CA), and phycoerythrin-conjugated anti-W6/32 (DakoCytomation, Glostrup, Denmark. Cells were fixed and permeabilized with 1.2% paraformaldehyde and 0.5% Tween 20, stained with fluorescein isothiocyanate-conjugated anti-p24 (Beckman Coulter, Fullerton, CA), and analyzed on an LSR II (BD Biosciences). After collecting 100,000 total cell events, percentages of marker-positive (CD4^+^, CD8^+^, and DP) thymocytes in the implant samples were determined by first gating on a live lymphoid cell population identified by forward- and side-scatter characteristics and then by CD3 expression. W6/32-positive mean fluorescence intensity (MFI) of DP thymocytes was determined, and CD4/CD8 ratios were calculated by dividing the percentage of CD4^+^ cells by the percentage of CD8^+^ cells for each individual implant.

### Statistical analysis

Results are expressed as the mean±SEM for each mouse group. Nonparametric statistical analyses were performed by use of the Mann-Whitney U test. Data for mice in each group were compared to those for untreated infected mice, and p values ≤0.05 were considered statistically significant.

## Results

### Viral load and prophylactic antiviral efficacy are highly reproducible among mouse cohorts each made with tissues from a single donor

**Figure 1:**
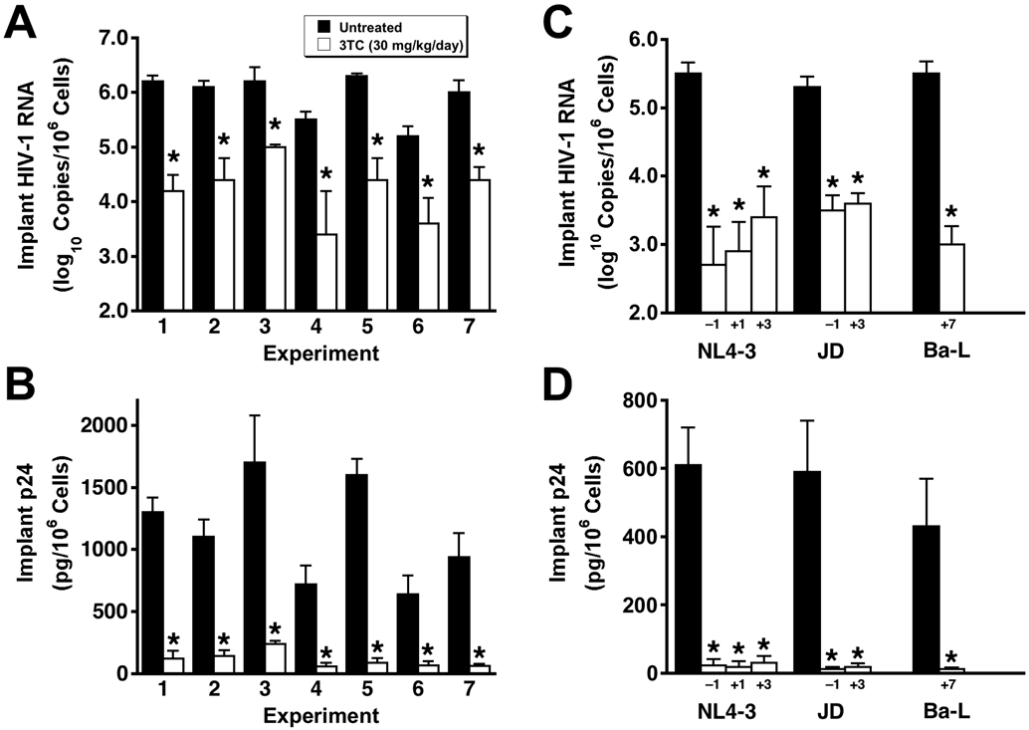
Implant viral loads in untreated- and 3TC-treated SCID-hu Thy/Liv mice are highly reproducible, and 3TC treatment after HIV-1 inoculation is nearly as effective as prophylactic treatment. A, HIV-1 RNA in Thy/Liv implants of untreated mice and mice treated by twice-daily oral gavage with 3TC at 30 mg/kg per day beginning 1 day before inoculation with NL4-3 (means±SEM) Each experiment was performed in a mouse cohort made with tissues from a single donor. B, Implant p24 for the same groups as in panel A. C, Implant HIV-1 RNA in mice treated with 3TC beginning on day −1, day +1, day +3, and day +7 with respect to inoculation with the indicated HIV-1 strains. D, Implant p24 for the same groups as in panel C. Implants were collected 21 days after inoculation for NL4-3, 14 days for JD, and 42 days for Ba-L. *p≤0.05, compared with untreated mice for 5–7 mice per group.

The levels of HIV-1 RNA and p24 in the Thy/Liv implants of untreated- and 3TC-treated SCID-hu Thy/Liv mice were highly reproducible among seven different mouse cohorts infected with the same stock of HIV-1 NL4-3 (Figure 1). In seven consecutive experiments, with mice in a given cohort constructed from human fetal tissue from a single donor, mean cell-associated HIV-1 RNA ranged from 5.2–6.3 log_10_ copies per 10^6^ cells (Figure 1A), and mean p24 ranged from 640–1,700 pg per 10^6^ cells (Figure 1B). There was also good correspondence between viral RNA and p24 means of implants in the same groups (i.e., lowest for untreated mice in experiment 6 and highest for those in experiments 3 and 5). In the seven experiments, 3TC treatment beginning 1 day before virus inoculation reduced HIV-1 RNA in the implants by 1.2–2.1 log_10_ (94%–99%) (Figure 1A) and p24 by 77%–92% (Figure 1B) compared to untreated mice in the same experiment.

Importantly, HIV-1 NL4-3 replication in these mice was genetically stable over the 21-day infection period; there was no evidence of the RT M184V substitution in viral RNA amplified by RT-PCR and sequenced from 32 of 32 Thy/Liv implants collected from mice treated with 3TC in these seven experiments (data not shown). The lack of resistance development is most likely the result of the limited time before virus-mediated thymocyte depletion becomes severe enough to limit the number of target cells available for infection. We view the genetic stability of the viruses over several weeks as an asset because our results are not confounded by the rapid development of antiviral resistance, particularly against drugs for which only one amino acid substitution is sufficient for high-level resistance.

### Postexposure dosing also reduces viral load and prevents virus-mediated thymocyte depletion

Initiation of 3TC treatment 1 or 3 days after NL4-3 inoculation was nearly as effective at reducing implant viral RNA (Figure 1C) and p24 (Figure 1D) as was prophylactic treatment with treatment beginning 1 day before inoculation. This was also true for mice inoculated with the clinical R5X4 isolate JD and treated with 3TC beginning 3 days after inoculation. Treatment of mice inoculated with the R5 isolate Ba-L, which replicates in Thy/Liv implants with slower kinetics than does X4 HIV-1 NL4-3 [16], was highly effective at reducing implant viral RNA and p24 even when initiation was delayed until 7 days after inoculation.

**Figure 2:**
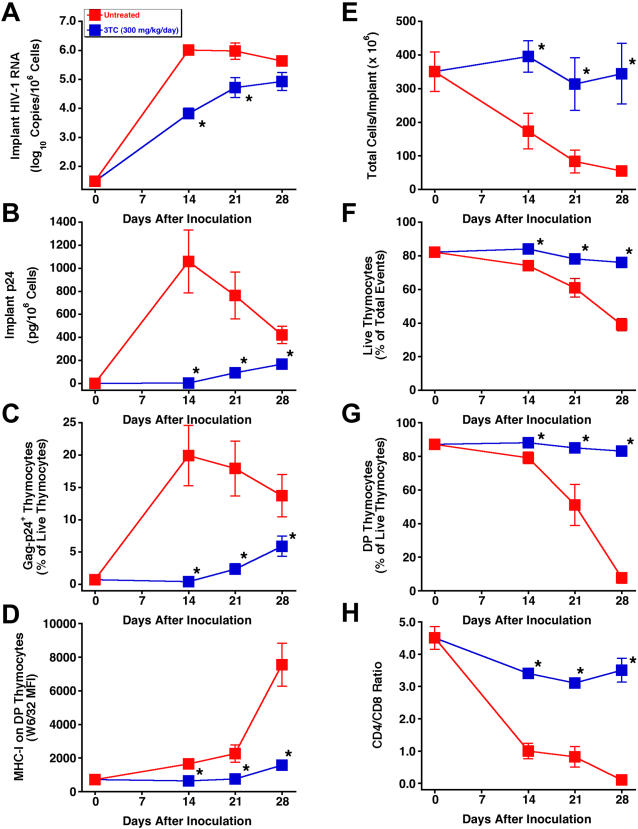
3TC treatment of HIV-1 JD-infected SCID-hu Thy/Liv mice inhibits viral replication and protects implants from virus-mediated thymocyte depletion. Mice were treated by twice-daily oral gavage with 3TC at 300 mg/kg per day beginning on day +1 after inoculation, and Thy/Liv implants were collected 14, 21, and 28 days after inoculation. Antiviral efficacy was assessed by determining HIV-1 RNA (A), p24 (B), Gag-p24^+^ thymocytes (C), and MHC-I expression on DP thymocytes (D). Thymocyte protection was assessed by total implant cellularity (E), thymocyte viability (F), percentage of DP thymocytes (G), and CD4/CD8 ratio (H) for 3TC-treated mice versus untreated mice (means±SEM). *p≤0.05, compared with untreated mice for 5–7 mice per group.

**Figure 3:**
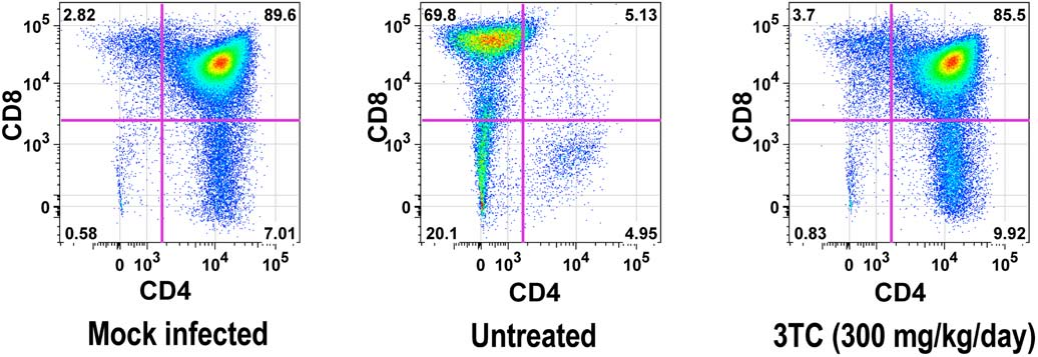
3TC treatment protects HIV-1 JD-infected Thy/Liv implants from thymocyte depletion. Flow cytometric analysis of representative implants stained for CD4 and CD8 obtained 28 days after inoculation with HIV-1 JD shows severe depletion of DP and CD4+ thymocytes in an untreated mouse and nearly complete protection from thymocyte depletion by 3TC treatment (300 mg/kg per day beginning on day +1 after inoculation).

To determine whether postexposure dosing also prevents thymocyte depletion, SCID-hu mice were inoculated with HIV-1 JD and treated twice daily with 3TC at 300 mg/kg per day in a time-course study in which implants were collected at weekly intervals up to 28 days after inoculation. By that point, the implants from untreated mice had become severely depleted of thymocytes. As expected, 3TC-treated mice had significant reductions in viral RNA (Figure 2A), p24 (Figure 2B), Gag-p24^+^ thymocytes (Figure 2C), and IFN-α-induced MHC-I expression on DP thymocytes (Figure 2D), compared to untreated mice at all three times. Reductions in viral load by 3TC were accompanied by virtually complete protection of the Thy/Liv implants from thymocyte depletion, in terms of total cellularity (Figure 2E), thymocyte viability (Figure 2F), percentage of DP thymocytes (Figure 2G), and CD4/CD8 ratio (Figure 2H). Flow cytometric analysis of representative implants obtained 28 days after inoculation with HIV-1 JD showed severe depletion of DP and CD4^+^ thymocytes in an untreated mouse and nearly complete protection from thymocyte depletion in one mouse treated with 3TC (Figure 3).

### Head-to-head comparisons of antiretroviral drugs at multiple dosage levels show relative potency

**Figure 4 pone-0000655-g004:**
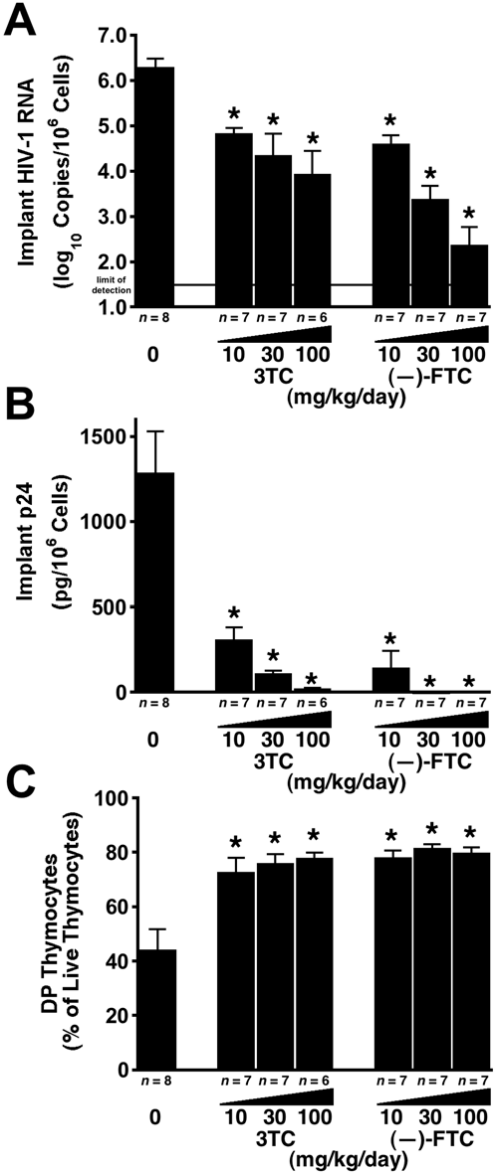
(–)-FTC is more potent than 3TC against HIV-1 NL4-3 in SCID-hu Thy/Liv mice. Mice were treated by twice-daily oral gavage with 3TC or (–)-FTC at 10, 30, and 100 mg/kg per day beginning on day –1. Antiviral efficacy was assessed by determining HIV-1 RNA (A) and p24 (B), and thymocyte protection was assessed by percentage of DP thymocytes (C) (means±SEM). *p≤0.05, compared with untreated mice for the number of mice indicated under each bar.

In the course of preclinical antiviral drug development, it is often important to determine whether a newly discovered agent is more potent than an existing, perhaps licensed, drug in the same class. To that end, we produced cohorts of SCID-hu Thy/Liv mice that were large enough for head-to-head comparisons of two drugs at multiple (typically three) dosage levels against one virus isolate. When the nucleoside analogs (–)-FTC and 3TC were compared, (–)-FTC was more potent at the same dosage levels, with greater reductions in viral RNA at 30 mg/kg per day (p = 0.035) (Figure 4A) and greater reductions in p24 at 10 mg/kg per day (p = 0.025) and 30 mg/kg per day (p = 0.009) (Figure 4B). The p24 levels in mice treated with (–)-FTC at 30 mg/kg per day were 1% that of mice treated with 3TC at the same dose (1,300 pg per 10^6^ cells in untreated mice versus 1.2 pg per 10^6^ cells for (–)-FTC and 110 pg per 10^6^ cells for 3TC) (Figure 4B). Good protection from thymocyte depletion was observed at all three dosage levels for both drugs (Figure 4C).

**Figure 5 pone-0000655-g005:**
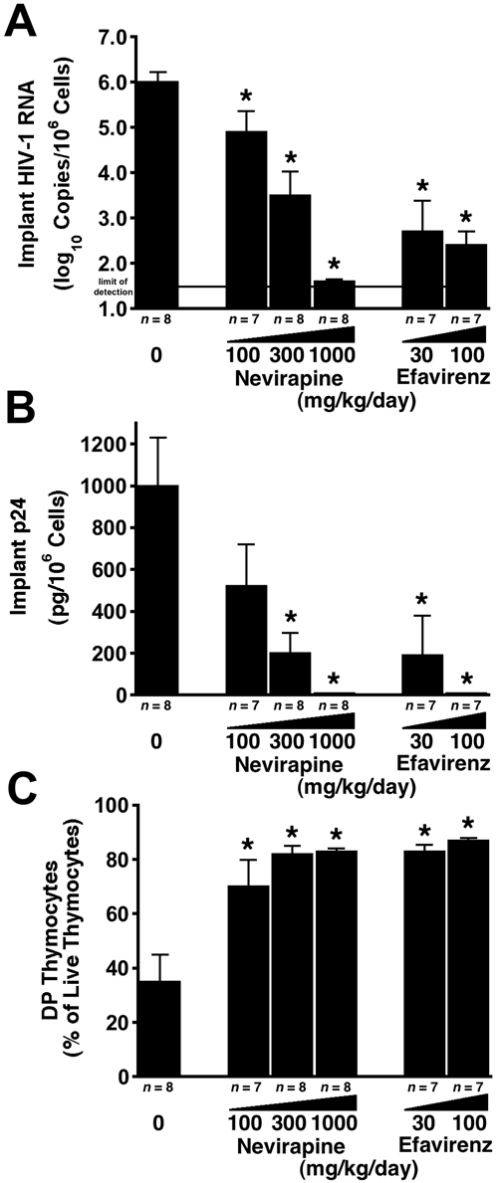
Efavirenz is more potent than nevirapine against HIV-1 NL4-3 in SCID-hu Thy/Liv mice. Mice were treated by twice-daily oral gavage with nevirapine or efavirenz at the indicated dosage levels beginning on day −1. Antiviral efficacy was assessed by determining HIV-1 RNA (A) and p24 (B), and thymocyte protection was assessed by percentage of DP thymocytes (C) (means±SEM). *p≤0.05, compared with untreated mice for the number of mice indicated under each bar.

In another experiment, the nonnucleoside RT inhibitor efavirenz was directly compared with nevirapine. Efavirenz was approximately 10 times more potent than nevirapine, as demonstrated by similar reductions in viral RNA (Figure 5A) and p24 (Figure 5B) and by thymocyte protection (Figure 5C) in groups treated with 300 mg/kg per day of nevirapine compared to 30 mg/kg per day of efavirenz.

**Figure 6:**
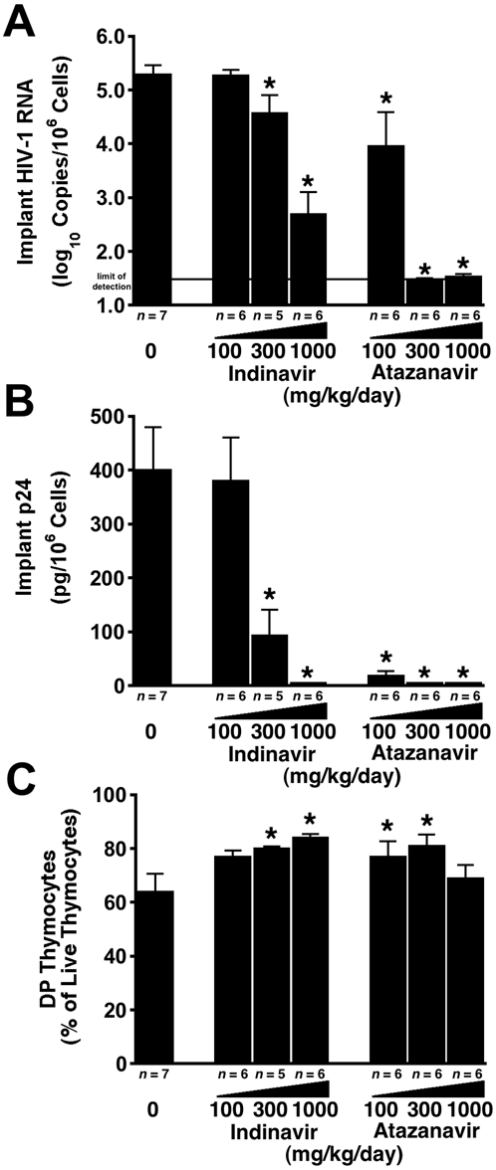
Atazanavir is more potent than indinavir against HIV-1 NL4-3 in SCID-hu Thy/Liv mice. Mice were treated by twice-daily oral gavage with indinavir or atazanavir at 100, 300, and 1000 mg/kg per day beginning on day −1. Antiviral efficacy was assessed by determining HIV-1 RNA (A) and p24 (B), and thymocyte protection was assessed by percentage of DP thymocytes (C) (means±SEM). *p≤0.05, compared with untreated mice for the number of mice indicated under each bar.

Finally, the second-generation protease inhibitor atazanavir was 3–10 times more potent than indinavir in NL4-3-infected SCID-hu mice, with a 3.1 log_10_ greater reduction in viral RNA (p = 0.006) with atazanavir than with indinavir at 300 mg/kg per day (5.3 log_10_ copies per 10^6^ cells in untreated mice versus <1.5 log_10_ copies per 10^6^ cells for atazanavir and 4.6 log_10_ copies per 10^6^ cells for indinavir) (Figure 6A). The p24 levels in mice treated with atazanavir at 100 mg/kg per day were 5% (p = 0.029) that of mice treated with indinavir at the same dose (400 pg p24 per 10^6^ cells in untreated mice versus 19 pg p24 per 10^6^ cells for atazanavir and 380 pg p24 per 10^6^ cells for indinavir) (Figure 6B). There was comparable protection from thymocyte depletion for the drugs at 100 and 300 mg/kg per day (Figure 6C).

### T-20 causes dose-dependent reductions in implant viral load

**Figure 7:**
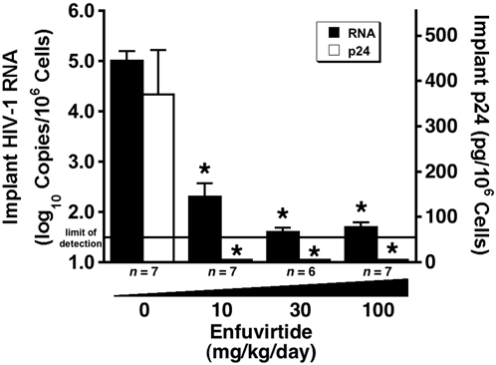
T-20 causes dose-dependent reductions in viral load in HIV-1 NL4-3 D36G-infected SCID-hu Thy/Liv mice. Mice were treated by twice-daily subcutaneous injection with T-20 at 10, 30, and 100 mg/kg per day beginning on day −1. Antiviral efficacy was assessed by determining cell-associated HIV-1 RNA and p24. Data are expressed as means±SEM; *p≤0.05 for treated mice versus untreated mice by the Mann-Whitney U test for the number of mice indicated under each bar.

Although we did not perform this experiment as a direct comparison with another HIV-1 fusion inhibitor, we are including these data to complete the interspecies scaling analysis described in the next section with a fourth class of antiretroviral (T-20). Treatment of NL4-3 D36G-infected SCID-hu Thy/Liv mice with T-20 by twice-daily subcutaneous injection resulted in dose-dependent reductions of both HIV-1 RNA and p24, reducing viral RNA by 3.3 log_10_ copies per 10^6^ cells and p24 to undetectable levels at 100 and 30 mg/kg per day (Figure 7).

### Inhibitory drug dosage levels are comparable between SCID-hu Thy/Liv mice and humans

The SCID-hu Thy/Liv mouse has the advantage of having human, not mouse, cells as the targets of antiretroviral uptake and action, but the pharmacokinetics of antiretrovirals in the mouse have the potential to be significantly different from those in humans. Small mammals usually eliminate drugs faster than large mammals [44], and toxic endpoints for therapeutics administered systemically to animals scale well between species when doses are normalized to body surface area [45]. To determine whether the doses found inhibitory in the SCID-hu Thy/Liv mouse bear any relation to those used clinically in humans, an interspecies scaling factor of 12.3 [46] was applied to generate mouse equivalent doses. This factor reflects the 12.3-fold difference in surface area-to-body weight ratio between mice (0.0066 m^2^/0.02 kg) and humans (1.6 m^2^/60 kg); consequently, 12.3 times more drug is required in the mouse to be comparable to the dose in humans. When currently approved dosage levels for humans of each antiretroviral drug are adjusted for this difference, the adjusted dosage levels were similar to those that cause 0.9–3.7 log_10_ reductions in viral RNA in the SCID-hu Thy/Liv mouse (Table 1). Thus, not only is the model predictive of relative potency within these drug classes, it might also be useful in determining approximate dosing ranges for effective use in humans.

**Table 1:** Antiretroviral drug dosage levels adjusted for difference in surface area-to-body weight ratio between mice and humans

Drug	Human dosage regimen^a^	Human dosage^b^ (mg/kg per day)	Mouse equivalent dosage^c^ (mg/kg per day)	Calculated reduction in implant HIV-1 RNA^d^ (log_10_ copies)
3TC	150 mg twice daily	5.0	62	1.9
(–)-FTC	200 mg once daily	3.3	41	2.3
nevirapine	400 mg once daily	6.7	82	0.9
efavirenz	600 mg once daily	10	123	3.7
indinavir	800 mg three times daily	40	490	1.2
atazanavir	400 mg once daily	6.7	82	1.4
T-20	90 mg twice daily	3.0	37	3.2

a From ref. [56].

b Based on 60 kg adult human weight and 20 g mouse weight.

c Human dosage×12.3 (fold difference in surface area to body weight between mice and humans; from ref. [45], [46].

d Calculated by linear regression of the viral RNA data shown in Figures 3–6 at the equivalent mouse dosage level.

### Multidrug-resistant NY index case HIV-1 replicates and depletes thymocytes with kinetics comparable to HIV-1 NL4-3

**Figure 8:**
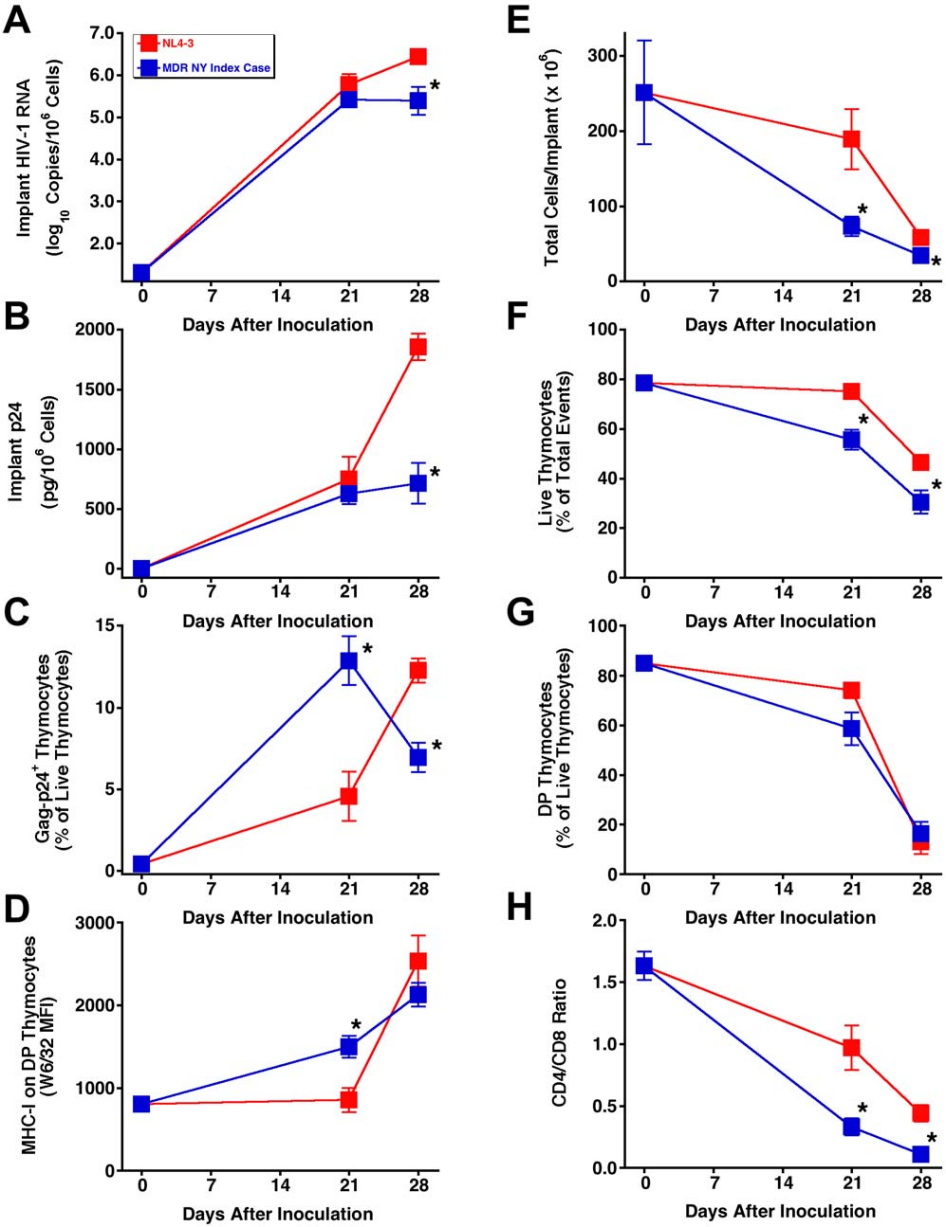
Multidrug-resistant (MDR) NY index case HIV-1 replicates and depletes thymocytes with kinetics comparable to HIV-1 NL4-3 in SCID-hu Thy/Liv mice. Viral replication assessed by determining HIV-1 RNA (A), p24 (B), Gag-p24^+^ thymocytes (C), and MHC-I expression on DP thymocytes (D). Thymocyte protection was assessed by total implant cellularity (E), thymocyte viability (F), percentage of DP thymocytes (G), and CD4/CD8 ratio (H) for NL4-3 versus MDR NY index case HIV-1-infected mice (means±SEM). *p≤0.05, compared with untreated mice for 7 mice per group.

A key test of any new class of antiretroviral drug is a demonstration of potent activity against HIV-1 with known drug-resistance mutations. We reported the activity of the 3TC analog dOTC against 3TC-resistant HIV-1 NL4-3/M184V in the SCID-hu Thy/Liv model [20]. Here we report that the MDR NY index case HIV-1, which was isolated from a patient with highly rapid progression to AIDS [43], replicates with kinetics similar to HIV-1 NL4-3 in SCID-hu Thy/Liv mice (Figure 8A–D) resulting in severe thymocyte depletion by 28 days after inoculation (Figure 8E–H). The lower viral loads for the NY index case isolate compared to NL4-3 at day 28 are most likely the result of somewhat faster thymocyte depletion and thus greater loss of target cells for viral replication.

Additional evidence that HIV-1 replication is genetically stable within the 28-day infection period was provided by sequence analysis of implant viral RNA showing that the MDR NY index case isolate [43] retained the full complement of drug-resistance amino acid substitutions of the original inoculum (data not shown). These data show that this MDR clinical isolate can readily be used as a challenge virus in the SCID-hu Thy/Liv model for *in vivo* studies of new classes of antiretroviral drugs with activity against HIV-1 that is resistant to currently licensed antiretrovirals.

## Discussion

The SCID-hu Thy/Liv mouse is an efficient model for the study of HIV-1 infection and for the preclinical evaluation of potential antiretrovirals. Here we show that this model provides a validated platform for reproducible viral infectivity and drug-mediated reductions in viral load *in vivo*. We also show that the model is especially valuable for direct, head-to-head comparisons of the potency and effective dose ranges of existing antiretroviral drugs. In consecutive experiments performed in different SCID-hu mouse cohorts infected with the same stock of HIV-1 NL4-3, we observed a range in mean HIV-1 RNA of only 1.1 log_10_ copies per 10^6^ cells. Reproducible reductions in viral load were also observed after oral administration of 3TC. Although our standardized protocol calls for prophylactic treatment 1 day before virus inoculation to maximize the potential for discerning antiviral efficacy of new drugs in initial *in vivo* tests, we show here that postexposure drug initiation (beginning 1, 3, or 7 days after HIV-1 inoculation) also renders potent antiviral efficacy. Importantly, potent therapeutic efficacy, in terms of both inhibition of viral replication and virtually complete protection from virus-mediated cytopathicity and thymocyte depletion, was also demonstrated in 3TC-treated mice infected with the highly cytopathic R5X4 isolate, JD. In addition, we have minimized one potential experimental variable by inoculating the mice at a uniform time point 18 weeks after implantation.

The goal of this study was to validate the SCID-hu Thy/Liv model for use in drug discovery efforts by determining the efficacy of different classes of FDA-approved antiretroviral drugs. Antiviral efficacy in this model is demonstrated by dose-dependent reductions in viral load as well as dose-dependent protection of CD4^+^ T cells from virus-mediated depletion. Assessment of thymocyte percentage and viability is particularly important for proving that the observed antiviral activity is not merely the result of cytotoxicity to the Thy/Liv implant just as the selectivity index (ratio of cytotoxic to inhibitory concentration) is important for assessing antiviral activity *in vitro*.

As new antiretroviral drugs in existing drug classes are developed to improve upon antiviral potency or to limit *in vivo* toxicity, direct comparisons of the existing drug and the new drug in single SCID-hu mouse cohorts represent a powerful preclinical tool for the prediction of improved efficacy in human patients. To accomplish this, we produced cohorts of SCID-hu Thy/Liv mice that were large enough for comparison of two drugs at three dosage levels against one virus isolate. For studies of viral pathogenesis and hematopoietic reconstitution, a relatively small number of animals can generate sufficient replicate points for analysis. In contrast, preclinical antiviral evaluations require groups of animals sufficiently large to demonstrate statistical proof of efficacy *in vivo*. Two-drug, three-dose comparisons are made possible by our unique ability to construct large (50–60-mouse) SCID-hu cohorts with tissues from a single donor, which permits six or seven dosing groups of 5–8 mice for optimal statistical power. Given such group sizes and appropriate controls, it is possible to assign effective antiviral dose ranges with statistical precision [8]. Among animal models for HIV-1, this ability is unique: nonhuman primate models (i.e., SIV- or SHIV-infected rhesus macaques) cannot provide dosing groups of such size and cannot achieve the same statistical power and relatively lower cost per animal. There are several recent reports on the infection of humanized Rag2^−/−^γc^−/−^ (RAG-hu) [47]–[50] and NOD/SCID BLT mice [51] with HIV-1, but there are no reports yet published on the evaluation of antiretroviral efficacy in these models, and it has not yet been demonstrated that 50–60 mice can be generated with the CD34^+^ hematopoietic stem cells from a single donor. In a recent review [52], Manz states that one of the challenges these CD34^+^-reconstituted mouse models face is that usually less than 10 mice can be transplanted from one human graft.

In the experiments presented here, the second-generation antiretrovirals in each drug class were more potent than their first-generation predecessors. Thus, (–)-FTC was more potent than 3TC, the nonnucleoside RT inhibitor efavirenz was more potent than nevirapine, and the protease inhibitor atazanavir was more potent than indinavir. The greater potency of the newer drugs in SCID-hu Thy/Liv mice is consistent with their observed greater potency in HIV-1-infected individuals. The nonnucleoside RT inhibitor (–)-FTC has greater potency than 3TC because the (–)-FTC-triphosphate is incorporated 10 times more efficiently than 3TC-triphosphate during HIV-1 RT-catalyzed RNA-dependent DNA synthesis [53]. The 10-fold greater potency of efavirenz compared to nevirapine in SCID-hu mice is entirely predictive of the superior oral bioavailability and plasma half-life characteristics of efavirenz [54], which allow for once-daily dosing in humans as the current standard of care compared with twice-daily dosing for nevirapine. Finally, the greater potency of atazanavir over indinavir in SCID-hu mice can be explained by a combination of its greater *in vitro* potency and oral bioavailability, allowing once-daily dosing [55]. Taken together, these comparisons of licensed antiretrovirals in each major class suggest that the SCID-hu Thy/Liv model may also predict clinical antiviral efficacy in humans. These results extend an earlier validation of the model with the nucleoside analogs zidovudine and dideoxyinosine [8]. The predictive nature of SCID-hu mouse efficacy is further supported by the dosage levels required to achieve log order reductions in implant viral load when adjusted for the difference in surface area-to-body weight ratio between mice and humans. Upon initial examination, the effective dosage level of 1,000 mg/kg per day for indinavir in the mice seems overly high until such an adjustment is made and the very high human daily dosage of 2.4 g (800 mg three times a day) is taken into consideration.

Predictiveness of the SCID-hu Thy/Liv model for the behavior of HIV-1 in humans can be extended beyond antiretroviral efficacy to simple kinetics of viral replication and virus-mediated cytopathicity. AZT-resistant (JD) and MDR NY index case R5X4 clinical isolates replicate and cause thymocyte depletion with kinetics comparable to HIV-1 NL4-3. The data presented here show that drug-resistant clinical isolates can readily be used as challenge viruses in the SCID-hu Thy/Liv model for *in vivo* studies of new classes of antiretroviral drugs with activity against HIV-1 that is resistant to currently licensed antiretrovirals.
